# Speculum-Induced Intraocular Pressure Elevation During Cataract Surgery and Its Association with Axial Length: A Retrospective Clinical Study

**DOI:** 10.3390/jcm15072520

**Published:** 2026-03-26

**Authors:** Hisaharu Suzuki

**Affiliations:** Zengyo Suzuki Eye Clinic, 1-22-11 Zengyo, Fujisawa 251-0871, Kanagawa, Japan; s5054@nms.ac.jp; Tel.: +81-466-0055; Fax: +81-466-0056

**Keywords:** eyelid speculum placement, intraocular pressure, cataract surgery

## Abstract

**Background/Objectives:** This study aimed to characterize eyelid speculum-induced intraocular pressure (IOP) elevation during cataract surgery and identify ocular biometric factors that stratify susceptibility to this pressure response. This study was conducted at Zengyo Suzuki Eye Clinic, Kanagawa, Japan. **Methods:** In this retrospective observational study, we analyzed 100 eyes that underwent routine cataract surgery. IOP was measured immediately before and within 10 s of speculum opening in the seated position using a rebound tonometer. The eyelid speculum was opened to a maximal opening position, and the opening width was recorded. Biometric parameters included axial length (AL), central corneal thickness, white-to-white distance, anterior chamber depth, and temporal angle-opening distance. Associations between IOP elevation and biometric factors were analyzed. IOP elevation rate was quantified as the percentage increase from baseline. The discriminatory performance of axial length was evaluated using receiver operating characteristic (ROC) analysis. **Results:** Overall, 100 patients (100 eyes) were included in the analysis. Mean IOP increased significantly from 15.75 ± 2.77 mmHg before speculum placement to 21.42 ± 5.54 mmHg after placement. The mean IOP elevation rate was 36.0 ± 27.4%. Shorter AL was consistently associated with a greater proportional IOP elevation. ROC analysis demonstrated consistent stratification of IOP elevation susceptibility by AL (area under the curve [AUC] = 0.645), with eyes shorter than 23.84 mm showing greater pressure elevation (sensitivity, 73.1%; specificity, 56.0%). Eyes in the upper quartile of the IOP elevation rate exhibited relatively greater pressure elevation. **Conclusions:** Eyelid speculum placement imposes a clinically meaningful IOP load during cataract surgery, with shorter ALs making eyes more biomechanically susceptible to IOP elevation.

## 1. Introduction

The stability of intraocular pressure (IOP) is a critical determinant of intraoperative safety in modern cataract surgery. IOP elevations associated with surgical maneuvers are typically transient; however, short-lived pressure spikes influence anterior chamber dynamics and ocular tissue stress, particularly in susceptible eyes. A clinically relevant source of IOP elevation is the mechanical force exerted by the eyelid speculum.

Previous experimental and clinical observations suggest that external periocular compression can alter IOP and the anterior segment configuration. However, the magnitude, inter-individual variability, and clinical implications of speculum-induced IOP elevation during routine cataract surgery have not been systematically characterized. Furthermore, the magnitude of the increase and patient-specific factors that predispose the eyes of certain patients to larger IOP spikes remain poorly defined [1,2,3,4,5].

External compression around the eye globe alters the anterior chamber dynamics. Experimental models have shown that periocular pressure induces a forward displacement of the posterior capsule and lens-iris diaphragm even when there is no intraocular fluidic instability [6]. These findings suggest that the mechanical forces transmitted through the eyelids may influence intraoperative ocular pressure. Because speculum-induced IOP elevation is not directly visible and is not specifically identified in phacoemulsification system readouts, substantial inter-individual variability in pressure response may remain unrecognized during routine procedures, even though modern phacoemulsification systems indirectly account for overall intraocular pressure changes.

Ocular biometric parameters influence the transmission of external forces to the globe. Eyes with shorter axial lengths (ALs) have a smaller ocular volume and higher scleral rigidity, which limits deformation and reduces the ability to dissipate external forces. Accordingly, such eyes may be biomechanically more susceptible to clinically meaningful IOP elevation in response to eyelid compression [7]. However, the association between ocular biometry and IOP elevation specifically induced by speculum placement has been examined in a few clinical studies. Understanding these relationships is important because surgeons routinely encounter unexpected anterior chamber shallowing or posterior capsule movement that fluidic settings alone cannot account for.

Modern phacoemulsification systems provide sophisticated pressure control and surge mitigation; however, speculum-induced IOP elevation is not directly measured or displayed during routine cataract surgery and may, therefore, go unrecognized when relying on system parameters or anterior chamber appearance alone. This hidden mechanical IOP load may be particularly relevant in eyes with low compliance or in patients at risk of pressure-related complications. Accordingly, identifying the preoperative biometric factors associated with exaggerated IOP elevation is necessary to improve perioperative safety in routine cataract surgery.

The aim of this study was to identify a simple preoperative anatomical marker that stratifies susceptibility to eyelid speculum-induced IOP elevation during cataract surgery.

## 2. Methods

### 2.1. Study Design and Participants

This retrospective observational study included repeat patients who underwent routine cataract surgery at the Zengyo Suzuki Eye Clinic between June and August 2022. Patients with glaucoma, prior ocular surgery, corneal pathology, uveitis, or intraoperative complications were excluded to minimize the confounding effects on baseline IOP and ocular biomechanics, thereby enabling a more accurate assessment of speculum-induced IOP changes under standardized conditions. The IOP measurements before and after eyelid speculum placement were routinely performed as part of the standard preoperative assessment in our clinic. The present study retrospectively analyzed the recorded data. Only the right eye of the participants was selected if both eyes were eligible. Overall, 100 eyes from 100 patients (45 males and 55 females; mean age of 75 years) met the criteria. The study was approved by the Institutional Review Board of the Zengyo Suzuki Eye Clinic (Approval No. 21000029) and was conducted in accordance with the tenets of the Declaration of Helsinki and the Ethical Guidelines for Medical and Biological Research involving human participants in Japan. Owing to its retrospective design, the Ethics Committee approved the use of an opt-out approach to collect patient data while ensuring participant anonymity. All patient data were anonymized prior to analysis by removing personal identifiers and assigning unique study identification numbers, ensuring that individual patients could not be identified.

### 2.2. IOP Measurements

All IOP measurements were performed preoperatively in an outpatient setting prior to cataract surgery. No intracameral anesthesia was administered at the time of IOP measurement. Topical anesthetic drops (0.4% oxybuprocaine) were routinely instilled before IOP measurement, following standard clinical practice. Mydriasis was induced using topical tropicamide 0.5% and phenylephrine hydrochloride 0.5% approximately 20 min before IOP measurement as part of routine preoperative preparation.

The patients were examined in a seated position and instructed to maintain steady fixation, relax the eyelids, and avoid squeezing the eyes during measurement. Eyelid manipulation and patient discomfort were carefully minimized during the IOP measurement, conducted immediately before and after eyelid speculum placement using a rebound tonometer (Icare; Icare Finland, Vantaa, Finland). These measurements do not represent the true intraoperative IOP during cataract surgery, which is performed in the supine position, but rather provide a controlled model for evaluating the relative IOP changes induced by external eyelid retraction. To ensure consistent corneal targeting, the patients were instructed to blink immediately before each measurement, and the probe was aligned with the central cornea. Each IOP value represented the mean of six consecutive readings.

A screw-type eyelid speculum (Leibinger; Blink Medical, Tuttlingen, Germany) was used in all cases. During insertion, the speculum was opened to its maximum width to standardize the external mechanical force applied to the eyelids. Subsequently, the actual opening width (mm) was measured immediately and recorded to assess the inter-individual variation in eyelid separation. This measurement was later evaluated to confirm minimal variability and verify that differences in opening width were unlikely to influence the observed variability in IOP elevation.

IOP elevation was defined as the numerical difference between the post- and pre-placement IOP. The IOP elevation was calculated as follows:

IOP elevation rate (%) = (IOPpost − IOPpre)/IOPpre × 100

The post-speculum IOP measurements were obtained within approximately 10 s of full speculum opening to ensure temporal consistency.

### 2.3. Ocular Biometric Parameters

Preoperative ocular biometry of AL was performed using an optical biometer (ARGOS, Alcon Vision LLC, Fort Worth, TX, USA). Other parameters collected included central corneal thickness (CCT), white-to-white distance (WTW), anterior chamber depth (ACD), and temporal angle opening distance (AOD), measured using anterior segment optical coherence tomography (CASIA 2 Advance, TOMEY, Nagoya, Japan).

### 2.4. Statistical Analysis

Continuous variables were assessed for normality and compared using paired *t*-tests, where appropriate. Associations between IOP elevation and individual biometric parameters were evaluated using Pearson’s and Spearman’s correlation coefficients.

The primary objective of this study was to examine the association between ocular biometric parameters and speculum-induced IOP elevation, focusing on identifying simple and clinically interpretable anatomical factors. Accordingly, univariate analyses were performed. Therefore, the potential influence of unmeasured or residual confounding factors was a limitation.

Scatter plots of IOP elevation and AL were generated to visualize the distribution of values. The primary outcome measure was the IOP elevation rate, defined as the percentage increase from the pre-speculum baseline IOP. The absolute IOP elevation (mmHg) was analyzed as a secondary descriptive measure to facilitate clinical interpretation.

### 2.5. Definition of High-Risk Eyes

For descriptive and exploratory purposes, eyes in the upper quartile (≥75th percentile) of the IOP elevation rate were categorized as exhibiting relatively greater speculum-induced IOP elevation (“high-IOP elevation” group). This definition was not intended to represent a clinically established risk threshold.

### 2.6. Receiver Operating Characteristic (ROC) Analysis and Optimal Cut-Off Determination

ROC analysis was performed as an exploratory assessment of the discriminatory performance of AL with respect to greater speculum-induced IOP elevation. ROC curves were constructed by calculating the sensitivity and specificity across various AL thresholds.

Because shorter ALs were associated with greater IOP elevations, AL values were multiplied by −1 so that higher values corresponded to greater IOP elevations in the ROC analysis. The transformation did not affect the ROC curve or the resulting cut-off value.

The area under the curve (AUC) was estimated using the trapezoidal rule. The optimal cut-off value was determined using the Youden Index (J = sensitivity + specificity − 1), which was calculated for each threshold. Sensitivity, specificity, and corresponding cut-off values were recorded.

This cut-off represented the AL threshold that best distinguished eyes exhibiting relatively greater IOP elevation (upper quartile) within the study cohort. All statistical analyses, including correlation analyses and ROC curve generation, were performed using Microsoft Excel (Microsoft 365, Redmond, WA, USA) with predefined spreadsheet templates to ensure consistency. Statistical significance was set at *p* < 0.05.

## 3. Results

Overall, 100 eyes from 100 patients were included in the analysis. The mean speculum opening width was 17.47 ± 1.29 mm, showing relatively limited inter-individual variation. The narrow distribution suggests that differences in eyelid separation alone may not fully account for the wide variability in IOP elevation, although no formal analysis was performed to assess the relationship between the opening width and IOP elevation.

### 3.1. Post-Speculum IOP Distribution

**Figure 1:**
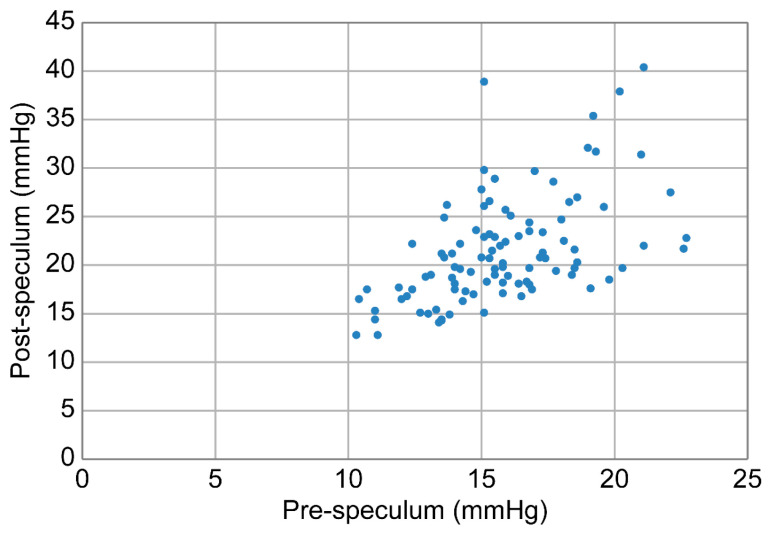
Scatter plot of post-speculum intraocular pressure (IOP) in all eyes (*n* = 100). Post-speculum IOP showed a broad distribution, with a small subset of eyes showing values approaching 40 mmHg when measured immediately after eyelid speculum placement in the seated position, indicating considerable inter-individual variability in the acute pressure response.

Figure 1 shows the distribution of the post-speculum IOP, demonstrating substantial variability, ranging from normal physiological levels to markedly elevated levels. A small subset of eyes exhibited post-speculum IOP values approaching 40 mmHg when measured immediately after eyelid speculum placement in the seated position, highlighting the substantial inter-individual variability in the acute pressure response.

### 3.2. Pre- and Post-Speculum IOP Comparison

**Figure 2:**
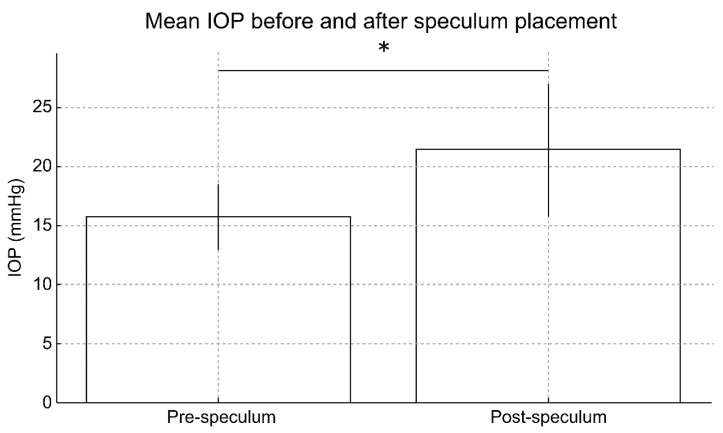
Mean IOP before and after eyelid speculum placement. Bars show mean pre- and post-speculum IOP with standard deviations (*n* = 100). IOP increased significantly from 15.75 ± 2.77 mmHg to 21.42 ± 5.54 mmHg after speculum placement (paired *t*-test, * *p* < 0.001).

The mean pre-speculum IOP was 15.75 ± 2.77 mmHg, whereas the mean post-speculum IOP increased significantly to 21.42 ± 5.54 mmHg (paired *t*-test, *p* < 0.001) (Figure 2). 

The magnitude of IOP elevation (post minus pre) varied widely among individuals, ranging from −1.5 to 23.8 mmHg, with a mean increase of 5.67 mmHg. The mean IOP elevation rate was 36.0 ± 27.4%.

### 3.3. Ocular Biometric Characteristics

Table 1 shows a summary of the ocular biometric characteristics of the study population, including AL, CCT, WTW, ACD, and AOD.

### 3.4. Correlation Analysis

**Figure 3:**
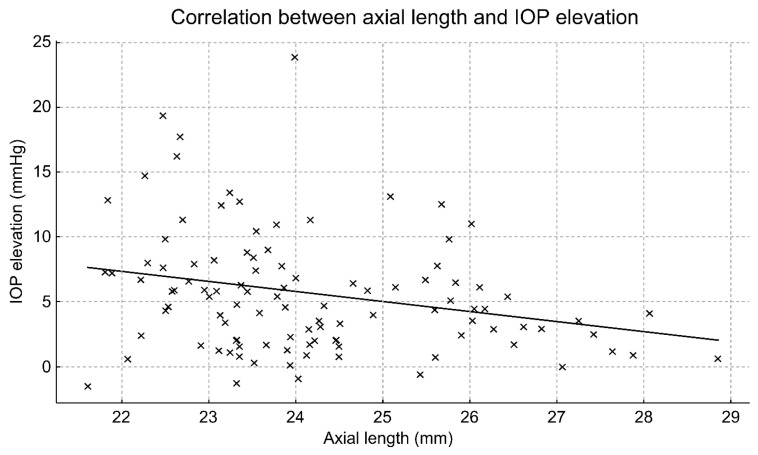
Correlation between the axial length (AL) and IOP elevation rate induced by eyelid speculum placement. The IOP elevation rate was defined as the percentage increase from the pre-speculum baseline IOP. A significant negative correlation was observed between AL and the IOP elevation rate (Pearson’s r = −0.27, *p* = 0.006; Spearman’s ρ = −0.255, *p* = 0.009), indicating that shorter eyes exhibited greater proportional IOP elevation than longer eyes.

Among all biometric variables examined, AL showed a significant negative correlation with the IOP elevation rate (percentage increase), indicating that shorter eyes exhibited a greater proportional IOP elevation (Figure 3) (Pearson’s correlation: r = –0.27, *p* = 0.006; Spearman’s correlation: ρ = –0.255, *p* = 0.009). These results indicate that shorter eyes are prone to greater IOP elevation following speculum placement. CCT, WTW, ACD, and temporal AOD were not significantly associated with IOP elevation (Table 1).

### 3.5. High-Risk Eye Definition

The 75th percentile IOP elevation rate was 51.9%. This value reflects the distribution of proportional IOP responses within the study population, rather than a predefined physiological or surgical threshold.

### 3.6. ROC Analysis of AL

**Figure 4:**
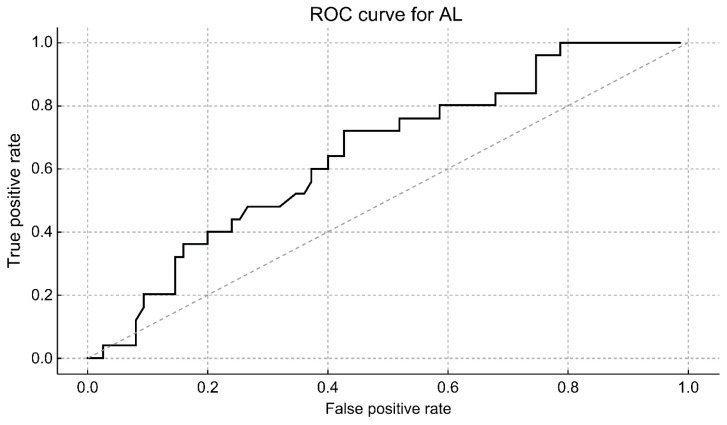
Receiver operating characteristic (ROC) curve illustrating the discriminatory performance of AL with respect to greater IOP elevation. Eyes with greater IOP elevation were defined as those in the upper quartile of IOP elevation rates. The area under the ROC curve (AUC) for AL was 0.645, indicating limited discriminatory performance. The optimal cut-off determined using the Youden Index was 23.84 mm, with a sensitivity of 73.1% and a specificity of 56.0% for distinguishing eyes with greater IOP elevation within the study cohort.

ROC analysis revealed limited discriminatory performance, with an AUC of 0.645 (Figure 4). Because shorter AL confers greater susceptibility to an increase in pressure, the negative AL value (−AL) was used as the predictor to ensure appropriate ROC directionality. High IOP elevation was defined as the upper quartile of the IOP elevation rate. The ROC analysis was performed using the IOP elevation rate as the outcome variable.

### 3.7. Optimal Cut-Off Determination

An AL threshold of 23.84 mm was associated with greater IOP elevation (sensitivity, 73.1%; specificity, 56.0%). Eyes below this threshold exhibited greater IOP elevation than those above the threshold during speculum placement.

## 4. Discussion

This study demonstrates that eyelid speculum placement imposes a clinically meaningful biomechanical IOP load during cataract surgery, with substantial inter-individual variability and a post-speculum IOP approaching 40 mmHg. Among the ocular biometric parameters examined, AL was the dominant anatomical determinant of susceptibility to speculum-induced IOP elevation; shorter eyes exhibited greater susceptibility. Other biometric factors, including CCT, WTW, ACD, and AOD, showed no meaningful correlations. An AL cut-off of 23.84 mm showed limited discriminatory performance in distinguishing eyes with greater IOP elevation.

Previous studies across different age groups and clinical settings have consistently shown that mechanical eyelid separation is associated with elevated IOP. Epley et al. reported an average increase of approximately 4 mmHg in children [1], whereas Çiçek et al. observed a mean increase of 2.7 mmHg in newborns undergoing routine eye examinations [2]. More recently, Iny et al. demonstrated that eyelid speculum placement is associated with significant IOP elevation in anesthetized pediatric patients, regardless of the speculum type [3].

Although direct comparisons are limited by differences in age, ocular biomechanics, anesthesia status, patient cooperation, measurement devices, and IOP assessment protocols, the consistent increase observed in IOP across age groups underscores the biomechanical nature of eyelid-induced ocular compression. Reportedly, rigid or widely opened specula may be associated with greater increases in IOP than wire-type instruments [4], potentially reflecting differences in the transmitted mechanical force. Because the eyelid force was not directly measured in this study, such mechanistic explanations should be interpreted hypothetically.

In addition to speculum mechanics, patient-generated external eyelid forces may also contribute to IOP elevation. Reportedly, forced eyelid closure or squeezing can substantially increase the IOP in awake patients and patients with open-angle glaucoma [5,8]. Such involuntary periocular muscle activation may occur to varying degrees during cataract surgery, particularly in patients with anxiety, and may include the pressure exerted by the speculum. The wide observed inter-individual variability in IOP responses suggests that other patient-related factors contribute to overall IOP elevation.

Notably, the magnitude of IOP elevation observed in this adult cohort was greater than that reported in previous pediatric or neonatal studies, which typically found an average increase of 2–4 mmHg [1,3]. This difference may partly reflect the lower scleral and corneal rigidity in pediatric eyes, which allows greater deformation under external mechanical forces. In this study, IOP elevation in many eyes increased by >5 mmHg, with a subset exceeding an increase of 10 mmHg. This larger IOP elevation likely reflects the combined effects of rigid speculum-induced eyelid tension and adult ocular biomechanics, as the rigid, widely opened speculum used likely exerted a more sustained eyelid retraction than the wire-type instruments commonly used in earlier studies. Furthermore, variable perioperative orbicularis muscle activity in awake adult patients may contribute to IOP elevation, potentially explaining the observed wide interindividual variability.

Posture also influences IOP, with higher IOP values reported in the supine position than in the seated position [9]. Because measurements in this study were obtained seated, the true intraoperative IOP during supine cataract surgery is likely higher than that measured in this seated model. Although this difference cannot be directly quantified, posture-related effects suggest that actual intraoperative IOP, particularly in shorter eyes, may be higher than that indicated by the present measurements.

Therefore, the greater pressure elevation observed in shorter eyes may reflect increased structural susceptibility to external compression. Although this could not be directly quantified, posture-related IOP behavior suggests that intraoperative pressure changes in short eyes may be underestimated. Further studies are required to clarify the interaction between posture and external forces during surgery.

A novel contribution of this study is the identification of AL as a meaningful determinant of susceptibility to externally induced IOP elevation. Eyes with shorter AL typically have reduced ocular volume and compliance, with relatively greater scleral rigidity [7], limiting their ability to deform under external mechanical load. Therefore, retracting the eyelid to an extent may result in a greater increase in IOP in short eyes than in longer, more compliant myopic eyes. This biomechanical vulnerability is consistent with the present findings and provides an explanation for the observed association between AL and IOP elevation. The primary analytical endpoint was the percentage increase in IOP, with the absolute IOP change reported for descriptive purposes.

Experimental evidence supports this concept. Using a slit-side view (SSV) porcine eye model, it was previously demonstrated that eyelid speculum compression significantly increased baseline IOP and induced pronounced posterior capsule elevation after an occlusion break, even with advanced surge-suppression technology such as the Active Sentry handpiece (Alcon Vision LLC, Fort Worth, TX, USA [6]). These findings indicate that externally transmitted forces can meaningfully alter intraoperative anterior chamber dynamics, providing mechanistic insight into clinical observations such as anterior chamber shallowing or posterior capsule billowing, despite stable fluidic settings.

Clinically, these results underscore the importance of minimizing external eyelid force, particularly in eyes with relatively shorter axial lengths. Potential strategies include limiting the speculum opening width, selecting low-tension designs, minimizing eyelid manipulation, and being aware of potential pressure elevation. Awareness of speculum-induced IOP elevation is important in settings requiring accurate IOP assessment.

Although the AUC was modest, AL consistently stratified susceptibility to speculum-induced IOP elevation; however, intraoperative IOP changes are multifactorial in nature, and AL represents only one of several structural parameters contributing to the eye’s biomechanical response to external compression, limiting its predictive performance.

Accordingly, the ROC-derived metrics should be interpreted as reflecting a consistent biomechanical susceptibility pattern. The upper-quartile classification was intentionally used as a data-driven approach to illustrate the relative susceptibility within the cohort and should not be interpreted to reflect a clinically meaningful risk category or harmful IOP threshold. Given the absence of confidence intervals, the observed AUC indicated limited discriminatory performance rather than robust discrimination.

## 5. Conclusions

This study has several limitations. It was conducted at a single center using a single-speculum design; dynamic intraoperative IOP fluctuations beyond the time of speculum insertion were not assessed. IOP was measured in the seated rather than supine position, possibly underestimating the absolute intraoperative IOP. The exploratory ROC analysis yielded a moderate AUC value of 0.645, indicating that although AL was the strongest associated biometric factor, additional unmeasured factors, such as eyelid tension, orbicularis muscle tone, patient-related factors, and scleral biomechanics, likely contributed to IOP elevation. Accordingly, the upper-quartile classification and ROC-derived cut-off should be interpreted as descriptive tools for characterizing the relative susceptibility within the cohort rather than as clinically validated risk thresholds. The longitudinal postoperative outcomes, including changes in the optic nerve and visual field, were not evaluated.

Despite these limitations, our findings demonstrate that eyelid speculum placement is a significant determinant of IOP elevation during cataract surgery. A short AL represents a readily identifiable anatomical parameter associated with a greater pressure response to eyelid retraction. Future studies incorporating continuous intraoperative IOP monitoring, advanced imaging modalities, and comparisons among different speculum designs may further clarify the mechanisms underlying pressure elevation and inform strategies for mitigating pressure-related complications during cataract surgery.

## Figures and Tables

**Table 1:** Ocular biometric characteristics of the study population.

Parameter (Unit)	Mean ± SD	Min	Max
AL (mm)	24.17 ± 1.61	21.61	28.85
CCT (µm)	537.96 ± 31.40	445	623
WTW (mm)	11.01 ± 0.90	10.27	12.10
ACD (mm)	2.66 ± 0.43	1.77	3.89
AOD (T) (mm)	0.42 ± 0.27	0.11	1.62

Values are presented as mean ± standard deviation (SD). AL, axial length; CCT, central corneal thickness; WTW, white-to-white distance; ACD, anterior chamber depth; AOD (T), temporal angle-opening distance.

## Data Availability

All data relevant to the study are included in the article.

## Abbreviations

IOP	Intraocular pressure
AL	Axial length
CCT	Central corneal thickness
WTW	White-to-white distance
ACD	Anterior chamber depth
AOD	Angle-opening distance
ROC	Receiver operating characteristic
AUC	Area under the curve
